# The Translational Future of Stress Neurobiology and Psychosis Vulnerability: A Review of the Evidence

**DOI:** 10.2174/1570159X21666230322145049

**Published:** 2023-03-27

**Authors:** Alexis E. Cullen, Javier Labad, Dominic Oliver, Adam Al-Diwani, Amedeo Minichino, Paolo Fusar-Poli

**Affiliations:** 1 Department of Psychosis Studies, Institute of Psychiatry, Psychology & Neuroscience, King’s College London, United Kingdom;; 2 Department of Clinical Neuroscience, Division of Insurance Medicine, Karolinska Institutet, Solna, Sweden;; 3 Department of Psychiatry, University of Oxford, Warneford Hospital, Oxford, United Kingdom;; 4 CIBERSAM, Sabadell, Barcelona, Spain;; 5 Department of Mental Health and Addictions, Consorci Sanitari del Maresme, Mataró, Spain;; 6 Early Psychosis: Interventions and Clinical-Detection (EPIC) Lab, Department of Psychosis Studies, Institute of Psychiatry, Psychology & Neuroscience, King's College London, United Kingdom;; 7 Department of Brain and Behavioural Sciences, University of Pavia, Pavia, Italy;; 8 OASIS Service, South London and Maudsley NHS Foundation Trust, London, United Kingdom;; 9 National Institute of Health Research Maudsley Biomedical Research Centre, South London and Maudsley NHS Foundation Trust, London, UK

**Keywords:** Schizophrenia, clinical high-risk, cortisol, cytokines, gut-microbiome, precision psychiatry

## Abstract

Psychosocial stress is a well-established risk factor for psychosis, yet the neurobiological mechanisms underlying this relationship have yet to be fully elucidated. Much of the research in this field has investigated hypothalamic-pituitary-adrenal (HPA) axis function and immuno-inflammatory processes among individuals with established psychotic disorders. However, as such studies are limited in their ability to provide knowledge that can be used to develop preventative interventions, it is important to shift the focus to individuals with increased vulnerability for psychosis (*i.e*., high-risk groups). In the present article, we provide an overview of the current methods for identifying individuals at high-risk for psychosis and review the psychosocial stressors that have been most consistently associated with psychosis risk. We then describe a network of interacting physiological systems that are hypothesised to mediate the relationship between psychosocial stress and the manifestation of psychotic illness and critically review evidence that abnormalities within these systems characterise high-risk populations. We found that studies of high-risk groups have yielded highly variable findings, likely due to (i) the heterogeneity both within and across high-risk samples, (ii) the diversity of psychosocial stressors implicated in psychosis, and (iii) that most studies examine single markers of isolated neurobiological systems. We propose that to move the field forward, we require well-designed, large-scale translational studies that integrate multi-domain, putative stress-related biomarkers to determine their prognostic value in high-risk samples. We advocate that such investigations are highly warranted, given that psychosocial stress is undoubtedly a relevant risk factor for psychotic disorders.

## 1 INTRODUCTION

Psychotic syndromes (most commonly, schizophrenia) include a diverse group of mental disorders characterised by distressing delusions and/or hallucinations (known as positive psychotic symptoms) and often accompanied by negative symptoms (*e.g*., affective flattening and anhedonia), cognitive impairment, and disorganised thinking and behaviour [1]. These disorders affect around 1% of the population during their lifetime [2] with a peak age at onset of 20.5 years [3] and a chronic course if sub-optimally treated [4]. Consequently, there has been a huge investment in understanding their complex aetiology and intervening during the early stages of illness, including preventive approaches [5].

Stress-vulnerability models of schizophrenia first emerged over 50 years ago [6, 7]. These models, which propose that psychosocial stress can contribute to psychosis onset among individuals with an underlying biological vulnerability for the disorder, have been hugely influential in shaping subsequent aetiological theories [8-14]. Whilst the fundamental principles of the stress-vulnerability model continue to have traction in both clinical and research settings, three major conceptual shifts have since occurred: First, our notion of what constitutes ‘vulnerability’ for psychosis has moved beyond one dominated by heritability, giving rise to new strategies for identifying individuals at increased risk for psychosis based on clinical features. Second, whilst early work in this field focused largely on major life events occurring proximal to illness onset, the range of psychosocial stressors that have been linked to psychosis has vastly expanded. Finally, increased consideration has been given to the neurobiological mechanisms that might underlie the relationship between stress and psychosis, particularly as such efforts might provide targets for intervention.

With regards to neurobiological mechanisms, there have been several excellent reviews on this topic [10, 15-19] which predominately focused on abnormal hypothalamic-pituitary-adrenal (HPA) function and immuno-inflammatory processes among individuals with established psychotic disorders and the influence of these systems on dopaminergic/glutamatergic function (two key neurotransmitters implicated in psychosis). Whilst the present article covers some of these topics, we shift the attention to the role of stress neurobiological mechanisms in promoting psychosis vulnerability. This is warranted given that studies of individuals with established psychosis are (i) retrospective in nature therefore cannot shed light on causality, (ii) confounded by antipsychotic medication and (particularly in populations with chronic schizophrenia) comorbid physical conditions and associated treatments, and (iii) limited in the extent to which they can provide knowledge that can be used to develop novel preventative interventions. In this critical review, we first describe the current strategies for identifying vulnerable individuals and psychosocial stressors that have been implicated in psychosis before providing an extensive review of hypothesised neurobiological mechanisms and interventions targeting these mechanisms, with a particular focus on evidence provided by meta-analyses (Tables **1**-**6**). After discussing these findings, we provide suggestions for how to move the translational field forward.

## 2 IDENTIFYING VULNERABLE INDIVIDUALS

### 2.1 Familial/Genetic High-Risk Strategy

Since the 1920’s, prospective studies throughout the world have followed the development of offspring and siblings of individuals with psychotic disorders from early life into adulthood [20]. This familial high-risk (FHR), or so-called, genetic high-risk strategy emerged based on early family studies demonstrating that schizophrenia is highly heritable [21]. Indeed, more recent studies indicate that the risk of developing schizophrenia is over seven times higher among offspring of individuals with the disorder [22]. Whilst genome-wide association studies have confirmed that schizophrenia has a substantial genetic component [23], as many offspring and siblings live with (or close to) their affected relatives, this increased risk likely reflects a combination of genetic and environmental factors. Although the FHR approach has been a highly successful research strategy, most individuals with schizophrenia do not have a relative with the disorder [24, 25], therefore risk factors identified in these studies may relate to a more familial form of illness. Moreover, recent cross-sectional studies often include adult relatives who have passed the age of peak risk for illness onset: As these relatives likely remain illness-free (despite having substantial genetic and environmental risk) due to protective factors, studying stress responsivity and neurobiological mechanisms in these older ‘high-risk’ populations has limited relevance for determining the prognostic value of these factors.

### 2.2 Schizotypal Personality Disorder and Traits

A further strategy for identifying individuals at increased risk for psychotic disorders is to study individuals who fulfil the criteria for schizotypal personality disorder (SPD) or who present high levels of schizotypal traits. SPD is a disorder within the psychosis spectrum that is characterised by attenuated forms of many of the traits and symptoms that define schizophrenia, most notably odd beliefs, speech, and behaviour. Whilst SPD symptoms can be distressing and pervasive, unlike those of schizophrenia, they do not typically confer the need for long-term care [26]. Nevertheless, individuals with SPD and those with high schizotypy traits have been found to share cognitive, neuroanatomical, and neurochemical features with individuals with schizophrenia [27]. Longitudinal studies show that around a third of individuals with SPD develop full-threshold psychotic disorder within 2-3 years [28, 29].

### 2.3 Clinical High-Risk Approach

The clinical high-risk approach, which focuses on help-seeking individuals who present features consistent with the prodromal phase of psychosis, has become the dominant high-risk strategy in the past twenty years [30]. Individuals who meet clinical high-risk for psychosis (CHR-P) criteria present attenuated psychotic symptoms, or (less commonly) brief, limited psychotic episodes, and/or trait vulnerability (first-degree relative with psychosis or fulfilment of SPD criteria) with functional decline; functional and neurocognitive impairments are also prevalent [31]. CHR-P individuals are typically detected in specialised, community-based clinics [32-35] and assessed with CHR-P specific psychometric interviews, which have excellent prognostic accuracy, although limited to group-level predictions [36]. The risk of transition to psychosis among CHR-P individuals varies over time, cumulating to 0.20 (95% CI, 0.19-0.21) at 2 years and 0.35 (95% CI, 0.32-0.38) at 10 years [37]. Transition risk also varies across CHR-P subgroups [38], being highest in those presenting with a brief, limited psychotic episode [39-42]. However, most of those who will not develop psychosis still present substantial mental health burden at follow-up [43, 44]. The prevalence of CHR-P features is around 10-fold higher in clinical samples (1.7%) than in the general population (19.2%) [45]. The combination of the relatively high risk for psychosis transition, with a low prevalence in the population, yields an associated global population attributable fraction of about 10% [46].

Given that the prodromal phase of psychosis is retrospectively reported by ~50% of first-episode psychosis (FEP) patients [47-50], the CHR-P strategy provides a unique opportunity to alter illness trajectory [51]. Assessment, treatment, and clinical monitoring of CHR-P individuals are now considered essential components of early intervention for psychosis in the UK [52]. Internationally, the CHR-P construct has been widely-implemented with 47 specialist CHR-P services providing care to over 20,000 CHR-P individuals worldwide [33]. These services aim to prevent the transition to full-threshold psychotic disorder [53], reduce the severity of attenuated positive and negative symptoms, and improve quality of life and functioning. Based on evidence that a longer duration of untreated psychosis (DUP, defined as the length of time between psychosis onset and the provision of adequate treatment) is associated with poorer clinical [54-58], cognitive [55, 59, 60], and social outcomes [55, 59, 61] among patients with psychosis, a further aim is to reduce the DUP for CHR-P individuals who do transition by facilitating prompt referral to FEP services. As such, CHR-P services can potentially improve outcomes after psychosis onset [62, 63].

### 2.4 Psychotic-like Experiences

Evidence emerging over the past decade indicates that psychosis exists on a continuum of severity, with members of the general population exhibiting “psychotic-like experiences” (PLEs) [64-66], typically defined as alterations in perception and reality that occur at the subclinical level [67]. These features are qualitatively different from the psychotic symptoms that characterise individuals at CHR-P and those with psychotic disorders [68] in that they are less commonly distressing and/or intrusive. PLEs are common in the general population, with an average lifetime prevalence of around 5% [69, 70], though estimates vary depending on the assessment method (*i.e*., self-report measures, which have questionable psychopathological validity, yielding higher prevalence estimates than interview-based assessments) and factors such age, sex, and country of residence. In youth, PLEs are more common in younger children than adolescents (median prevalence ~17 *vs*. 8%, respectively [71]), with longitudinal studies showing that most PLEs experienced during childhood and early adolescence are transient [72-75]. Although the vast majority of individuals who experience PLEs are not help-seeking, children and adolescents who report PLEs are nearly four times more likely to develop psychotic disorders and three times more likely to develop any mental disorder [76], although the absolute risk is so low that preventive interventions are infeasible. Furthermore, PLEs (at any age) appear to confer an increased risk for suicidal ideation and suicide attempts [77]. Given the potential to recruit large samples from the general population (which are often free from medication and other confounds), the PLE strategy has been widely utilised in studies examining stress neurobiology. However, it is important to note that PLEs are qualitatively different from psychotic symptoms and disorders and cannot be used as a clinically valid proxy indicator [78].

## 3 PSYCHOSOCIAL STRESSORS

Psychosocial stressors occurring throughout the lifespan have been associated with psychotic symptomatology. This section considers the psychosocial stressors most-commonly examined in studies of individuals with psychosis and high-risk populations.

### 3.1 Childhood Adversity

Childhood adversity is a broad term encompassing psychosocial stressors experienced throughout childhood and early adolescence, which can include trauma/maltreatment, bullying, and parental death. These experiences have been robustly associated with the onset of psychosis (OR = 2.87, 95% CI: 2.07 to 3.98) [79-83], even when restricted to prospective studies (OR = 2.52, 95% CI: 1.27 to 5.02) [80]. Meta-analytic evidence indicates that emotional abuse confers the highest risk for psychosis (OR = 3.40, 95% CI: 2.06 to 5.62), followed by physical abuse (OR = 2.95, 95% CI: to 2.25 to 3.88), sexual abuse (OR = 2.38, 95% CI: 1.98 to 2.87), bullying (OR = 2.39, 95% CI: 1.83 to 3.11), neglect (OR = 2.90, 95% CI: 1.71 to 4.92), and parental death (OR = 1.70, 95% CI: 0.82 to 3.53) [79]. Importantly, childhood adversities are a transdiagnostic risk factor for several mental disorders and the one with the largest preventive capacity: For example, a recent meta-analysis [46] determined that reducing childhood adversities would decrease the incidence of schizophrenia-spectrum disorders by 37.84% (global population attributable fraction 95% CI: 26.77% to 48.40%).

With regards to FHR studies, childhood trauma has been associated with subclinical psychotic symptoms in both siblings of patients with psychosis and healthy controls [84], suggesting that this association may be independent of underlying familial risk. CHR-P individuals are more likely to report childhood trauma than healthy controls (OR = 2.90, 95% CI: 2.90 to 12.2) [85]; which appears to be driven by higher rates of emotional abuse (OR = 5.84, 95% CI: 1.79 to 19.03) and physical neglect (OR = 3.07, 95% CI: 1.04 to 9.01), with no significant differences in other childhood trauma domains [85]. Childhood adversity also increases healthcare costs in CHR-P individuals [86], as well as the likelihood of presenting other comorbid mental disorders (including depression, PTSD, panic disorder and social phobia) [87]. Whilst meta-analyses have shown no association between childhood trauma and transition to psychosis in CHR-P individuals [88], childhood trauma has been found to increase risk of subsequent PLEs in general population samples and mediate the relationship between perinatal risk factors and these symptoms [89].

### 3.2 Stressful Life Events

Experiencing stressful life events in adulthood, such as job loss, serious illness, divorce, death of a loved one, or victimisation, substantially increases psychosis risk in the general population (OR = 5.34, 95% CI: 3.84 to 7.43) [80], an effect that has been confirmed in prospective studies [90]. As these events can occur during a wider time-frame than childhood adversity, there is heterogeneity across studies in the time-period investigated, with some studies examining events in the past three months [91], and others including events occurring over several years [90]. Early evidence from small studies suggested that stressful life events tended to increase in the three weeks prior to the onset of psychotic symptoms [91] but this has not been replicated [92].

With regards to high-risk samples, a study that included children at FHR and those presenting PLEs (alongside other antecedents of schizophrenia) observed that both groups reported a greater number of negative life events than their typically-developing peers [93]. Moreover, the PLE group experienced a higher frequency of daily stressors than typically-developing children and were more distressed by these experiences. Similarly, data from the North American Prodrome Longitudinal Study (NAPLS-2) suggests that CHR-P individuals are more likely to experience both stressful life events and daily hassles than healthy controls, and report greater subjective distress in relation to these events [94, 95]. However, a subsequent study from the NAPLS-2 cohort found that neither stressful life events nor childhood trauma were independently associated with transition status at 1-2 years in multivariable analyses and that these variables made little contribution to the predictive models [96]. Thus, psychosocial stressors may have a limited ability to predict transition in CHR-P youth over and above baseline symptoms and demographic factors, perhaps because they are highly prevalent in this population.

### 3.3 Momentary Stress

Stress is a dynamic experience that fluctuates throughout the day, but these daily changes are difficult to assess retrospectively with clinical interviews or self-report questionnaires [97]. Advances in technology have allowed for repeated and momentary evaluation of stress and symptomatology, referred to as either experience sampling methods (ESM [98]) or ecological momentary assessment (EMA [99]). These methods typically assess stress associated with momentary activities using Likert scales, which the user is promoted to complete at randomly-timed intervals throughout the day. Many devices also include items capturing self-reported psychotic and depressive symptoms, which have been shown to correlate with standard researcher-assessed clinical instruments [100]. Consequently, there has been recent interest in using EMA devices in clinical settings to facilitate remote monitoring of symptoms and illness [101].

Early ESM studies reported that patients with psychotic disorder and FHR individuals exhibited higher levels of emotional stress reactivity (defined as a decrease in positive affect and an increase in negative affect in response to stress) than healthy controls [102]. Moreover, increases in activity-related stress were associated with greater intensity of psychotic symptoms (referred to as psychotic stress reactivity) in patients and relatives but not controls [103]. Subsequent ESM studies have similarly observed that CHR-P individuals show increased emotional stress reactivity compared to healthy controls [104] and that the magnitude of emotional stress reactivity is associated with transition to psychosis [105]. Consistent with these findings, a large study in the general population observed that both baseline emotional and psychotic stress reactivity were associated with the persistence of PLEs over a 14-month follow-up [106].

### 3.4 Sociodemographic Factors

Sociodemographic factors, such as ethnic minority and migrant status, are robustly associated with psychosis onset [80], an effect that is likely attributable in part to psychosocial stress [107]. For example, being a first-, second- or third-generation migrant is associated with higher rates of stressful life events and trauma and low sociodemographic status [108-110], whilst perceived racial discrimination increases the severity of depression, anxiety, and stress as well as PLEs [111]. In contrast to studies of patients with established psychosis, CHR-P individuals are not more likely to be ethnic minorities compared to healthy controls [112]. Moreover, non-white ethnicity does not appear to increase psychosis risk in CHR-P populations [113], although there is some evidence that ethnic minority CHR-P individuals experience greater exposure to trauma [114]. Similarly, CHR-P individuals are less likely to be first-generation immigrants compared to healthy controls [115] and the evidence for an effect of migrant status on transition is mixed [116]. This discrepancy between ethnicity and migrant status increasing the general population’s risk for psychosis, but having limited effect within CHR-P populations, could suggest differences in illness progression and/or sub-optimal identification of at-risk individuals in these population groups. Individuals from ethnic minority and/or migrant backgrounds may have a more acute progression of illness with a relatively short prodrome, making early detection more difficult. Indeed, migrant status is associated with delays in help-seeking and a longer duration of untreated psychosis [117, 118], potentially related to a lack of understanding of local service provision [119], difficulty communicating in a non-native language [120], and low mental health literacy [120]. Together, these elements could result in delayed detection in these vulnerable populations. Living in an urban environment is likewise robustly associated with developing a psychotic disorder [80] but not with a transition to psychosis in CHR-P individuals [113]. Conversely, neighbourhood level of social deprivation, however, does not appear to affect psychosis risk in the general population [80] but is related to PLEs [121].

### 3.5 Interaction with Substance Use

Substance use has become an increasingly important consideration for psychosis risk, with cannabis [80, 122], tobacco [80] and novel psychoactive substances [123] (including synthetic cannabinoids) all shown to increase the risk for psychotic disorders. This has been echoed by an increased incidence of substance-induced psychoses in recent years [124, 125]. In line with neurodevelopmental models, both prenatal and postnatal substance use exposure has been associated with an increased risk of psychosis [126]. While some of the variability in risk may be explained by age at first use, frequency of use, and genetic factors [122, 127], psychosocial stress exposure may also influence the extent to which substance use contributes to emerging psychopathological states [128, 129]. For example, evidence suggests that childhood trauma and cannabis use interact to increase the risk and frequency of PLEs in adolescents and young adults [130, 131].

## 4 NEUROBIOLOGICAL MECHANISMS

In the next section, we discuss a network of interacting physiological systems that are hypothesised to mediate the relationship between psychosocial stress and the manifestation of psychotic illness.

### 4.1 HPA Axis

The neural diathesis-stress model, proposed in 1997 by Walker and Diforio [9] and subsequently updated [8, 10], was the first to suggest that the HPA axis may mediate the relationship between stress and psychosis. In mammalian organisms, the physiological stress response is coordinated by the sympathetic branch of the autonomic nervous system (triggering a rapid release of adrenaline which has immediate effects on heart rate, vasoconstriction, and digestion), and subsequently, the HPA axis [132, 133]. Of the two systems, the HPA axis has received particular attention from psychopathologists. In brief, the HPA axis responds to stress by eliciting a cascade of hormonal reactions (release of corticotrophin-releasing hormone (CRH) from the paraventricular nucleus of the hypothalamus and subsequently adrenocorticotropic hormone (ACTH) from the pituitary), resulting in the secretion of glucocorticoids from the adrenal cortex. Glucocorticoids (in humans, primarily cortisol) facilitate behavioural and physiological responses to stress by interacting with various systems to influence glucose metabolism, and immune, cardiovascular, and brain function [8]. The brain expresses two forms of the glucocorticoid receptor: high-affinity mineralocorticoid receptors (MRs), which are occupied at rest, and low-affinity glucocorticoid receptors (GRs) that are typically occupied only under high glucocorticoid conditions [133]. Glucocorticoids regulate HPA axis activity by binding to these receptors in the hypothalamus and pituitary where they inhibit the secretion of CRH and ACTH respectively *via* negative feedback [134].

HPA activity can be inferred from levels of cortisol and ACTH in bodily fluids. Given that cortisol can be measured in blood, urine, saliva, and (more recently) hair, it has become the most commonly-used HPA axis measure in research settings. Cortisol can be measured under basal (*i.e*., unstimulated) conditions, in response to awakening, following exposure to experimental psychosocial stressor tasks or pharmacological challenge, and (using the ESM approaches described above), in response to naturally occurring stressors (for a comprehensive review of cortisol measures see [10]). Pituitary volume has also been employed as a measure of HPA axis function, albeit less commonly: enlargements are thought to reflect an increase in the size and ratio of ACTH-producing corticotroph cells within the anterior pituitary [135], thus indicating increased HPA axis activity. Given that cortisol levels fluctuate substantially throughout the day, pituitary volume may provide a more stable HPA axis measure [136].

#### 4.1.1 Cortisol Levels in High-risk Populations

Meta-analyses (Table **1**) indicate that individuals with psychotic disorders are characterised by increased levels of unstimulated blood and salivary cortisol compared to healthy controls [137-139], but an attenuated/blunted cortisol response during psychosocial stressor tasks and after awakening [140-143]. It has been proposed that this pattern reflects repeated exposure to psychosocial stressors (leading to chronically elevated cortisol levels) that, when exhausted, gives rise to a dysregulated system that is unable to mount a sufficient response to acute stressors [19]. Consistent with this hypothesis, a small study measuring hair cortisol levels (indexing chronic cortisol secretion) observed higher levels among 27 medication-naïve FEP patients relative to controls [144], although a smaller study of 16 patients with schizophrenia (including 10 FEP) failed to confirm these findings [145].

Meta-analyses of cortisol levels in high-risk populations (Table **1**) have, however, yielded inconsistent results. In the first meta-analysis to examine salivary cortisol levels in CHR-P individuals [146], pooled data from eight studies indicated a significant increase relative to controls. However, a more recent meta-analysis [138] observed no significant differences in unstimulated salivary cortisol in CHR-P individuals relative to controls (Hedges' g = 0.15, 95% CI: -0.01 to 0.31) and no differences in blood cortisol levels (Hedges' g = 0.39, 95% CI: - 0.42 to 1.21). Moreover, neither of these meta-analyses observed differences in basal salivary cortisol levels when comparing CHR-P individuals who subsequently transitioned to psychosis and those who did not [138, 146]. With regards to dynamic cortisol measures, two meta-analyses, including three [146] and five studies [138], respectively, both observed that the cortisol awakening response (CAR) was blunted, but not significantly so. However, both reviews pooled data across diverse high-risk populations. Whilst no meta-analysis has yet examined cortisol responses during psychosocial stressor tasks, significantly blunted responses relative to controls have been observed in studies of CHR-P individuals [147] and those with high schizotypy traits [148], but not in adult FHR populations [149]. Similarly, the few studies that have examined hair cortisol in high-risk populations have yielded inconsistent results, with one study reporting increased hair cortisol among CHR-P youth [150] but a subsequent study finding no differences between high-risk individuals (CHR-P and FHR combined) and controls [151].

More recently, it has been shown that incorporating salivary cortisol levels in multivariable models can improve prediction of illness progression in high-risk samples. An analysis of data from the NAPLS-2 cohort showed that the inclusion of basal salivary cortisol measured at baseline in a multivariable model (which included baseline positive symptoms and functioning) significantly improved prediction of transition status [152]. Consistent with this finding, in a cohort of children enriched for psychosis risk factors, diurnal cortisol levels at age 11-14 years predicted development of attenuated psychotic symptoms at age 17-21 years in a multivariable model that included demographic factors and baseline psychopathology [153]. Whilst these findings require replication, they indicate that further research into the predictive utility of salivary cortisol in psychosis high-risk groups is warranted, a topic that we will discuss in Section 6.

#### 4.1.2 Pituitary Volume in High-risk Populations

While an early narrative review concluded that the onset of psychosis was associated with pituitary volume enlargement (independent of antipsychotic use) followed by a reduction in volume during chronic illness [135], subsequent studies have been inconsistent. A meta-analysis of nine studies (Table **1**) observed no significant difference in pituitary volume when comparing patients with established psychosis (schizophrenia and FEP) and healthy controls [154], although a trend for larger volume was observed in the FEP group. In this review, effect sizes were positively correlated with the proportion of individuals receiving antipsychotic medication. With regards to high-risk populations, this same meta-analysis observed no differences in pituitary volume when all high-risk individuals (CHR-P and SPD) were compared with controls [154], although increased volume was reported in those who transitioned (SMD = 0.37, 95% CI: 0.00 to 0.75). In a subsequent meta-analysis [155], a significant increase in pituitary volume was observed in high-risk individuals when data from 11 heterogenous samples were pooled (Hedges' g = 0.16, 95% CI: 0.00 to 0.32), although sub-analyses performed for FHR and CHR-P groups showed no differences to controls. Moreover, individuals who later transitioned to psychosis showed even larger increases in pituitary volume (Hedges' g = 0.55, 95% CI: 0.06 to 1.04) and consistent with the previous meta-analysis, larger effect sizes across all studies were positively associated with the proportion of individuals receiving antipsychotic medication. As was the case with salivary cortisol, the largest study included in this review found that pituitary volume was a significant predictor of transition to psychosis in CHR-P individuals when included in a multivariable model accounting for baseline symptoms [156].

#### 4.1.3 Concordance with Stress Measures

The neural diathesis-stress model posits that individuals with increased vulnerability are more sensitive to the effects of psychosocial stressors due to abnormalities within the HPA axis. As such, we might expect individuals on the psychosis spectrum to exhibit abnormal HPA axis responses to psychosocial stressors. Whilst this has been shown to some extent in laboratory studies using psychosocial stressor tasks (where individuals with established illness and some high-risk groups have been found to exhibit hypo-responsivity), far less is known about cortisol responses to the naturally-occurring psychosocial stressors that have been associated with psychosis risk.

Addressing this knowledge gap, a recent meta-analysis (Table **1**) pooled data across studies examining the concordance between naturally-occurring psychosocial stressors and cortisol in individuals with established psychosis, high-risk individuals (CHR, FHR, SPD, PLEs), and healthy controls [157]. Analysis of 134 effect sizes from all three populations indicated that psychosocial stressors and cortisol measures were only weakly correlated (*r =* 0.05, 95% CI: -0.00 to 0.10) and that neither established psychosis status (*r =* 0.01 *p* = 0.838) nor high-risk status (*r =* 0.02, *p* = 0.477) had a significant effect of the strength of correlation. Thus, there was no evidence to suggest that high-risk individuals or those with established psychosis exhibited either hypo- or hyper-responsivity to naturally-occurring psychosocial stressors. However, this review identified several major limitations with the extant literature, including, small samples, lack of adjustment for potential confounders, and failure to account for (or report) the time-lapse between stressor measurement and cortisol collection. The latter appears to be particularly important: in a further study from the NAPLS-2 consortium (n = 662), the degree of concordance between psychosocial stressors and basal cortisol was found to be moderated by the lapse-of-time between collection of these measures, where daily stressors, life events, and childhood trauma were only associated with basal cortisol when these stress measures were completed on the same day as cortisol collection [95]. After accounting for the lapse-of-time between assessments, the degree of concordance was found to be stronger among CHR-P individuals who later converted to psychosis when compared to those who did not [95]. These findings concur with those from studies using ESM to examine cortisol responses to daily stressors (where cortisol measures are obtained within 10 minutes of rating stressful experiences). Indeed, two ESM studies, which could not be included in the meta-analysis due to incomparable effect sizes [158, 159], reported that event stress was correlated with salivary cortisol in individuals with psychosis and FHR groups but not in healthy controls. Further studies, that address the methodological issues identified in this recent meta-analysis, are required to determine whether stressor-cortisol concordance is a useful marker of psychosis risk.

### 4.2 Immuno-inflammatory Processes

There is growing interest in integrating immune and inflammatory mechanisms within diathesis-stress models of psychosis risk for diagnostic, prognostic, and therapeutic purposes [160]. Alongside the HPA axis, the immune system helps maintain homeostasis in response to acute and chronic stressors; indeed, cortisol is a potent regulator of immune activity [161]. In brief, the immune system consists of molecular and cellular components. Cytokines are the key signalling molecules, which, like other hormones, can signal at short distances between cells but also systemically, coordinating physiological responses. Cellular components include B and T lymphocytes with receptors to specific antigens and non-lymphocytic cells such as neutrophils, macrophages, and dendritic cells. These are organised functionally into innate and adaptive arms: the former is evolutionarily ancient and mediates broad and non-specific responses to infection and other physiologic challenges by promoting inflammation (which acts to clear the challenge and prime adaptive immunity), whilst adaptive immunity mediates antigen-specific responses *via* humoral (antibody) and cellular mechanisms. Acute inflammation is usually useful and adaptive, turning off once resolved, whereas chronic inflammation is typically maladaptive and associated with tissue-specific or systemic pathology. The most commonly-studied immune markers in psychosis research are peripheral cytokines and markers of chronic low-grade inflammation (*e.g*., C-reactive protein) [162, 163]. Although there has been recent interest in clotting and complement proteins [164] and neuronal surface auto-antibodies [165].

#### 4.2.1 Early Life Stress and Inflammatory Markers

Converging evidence suggests that the immune system may mediate the effect of early life stress on neurodevelopment and psychosis risk. Studies using rodent maternal immune activation (MIA) models show that offspring of pregnant mice subjected to peripheral inflammatory stimuli exhibit structural, functional, and behavioural features relevant to psychosis risk, including changes in ventricular size, pre-pulse inhibition, HPA axis sensitivity, and locomotive and social behaviour [166]. In humans, early-life stress has been shown to influence immune set-points and response to inflammatory challenges. For example, young children from families with high psychosocial stress were found to show low spontaneous immune activity as indexed by peripheral cytokines when compared to children from low-stress families, but an increased immune response when cells were stimulated *in vitro* [167]. As children in the high-stress group were also characterised by elevated cortisol, these findings suggest that psychological stress can influence immune system parameters and HPA axis markers. In terms of psychosis risk, an analysis of data from the ALSPAC study (a prospective longitudinal cohort of children from the general population) observed that higher levels of IL-6 measured at age 9 were associated with psychotic experiences and disorder at age 18 [168]. However, this effect was not disorder-specific, as risk of depression was also elevated. In the same cohort, proteomic analysis indicated that elevations in complement proteins at age 12 were associated with risk of psychotic experiences at age 18 [169].

#### 4.2.2 First Episode Psychosis and State Markers

Meta-analyses (Table **2**) indicate that medication-naïve FEP patients are characterised by elevations in several cytokines (*e.g*., IFN-γ, IL-6, IL-17, TGF-β, and TNF-α) [170-172]. Whilst it has been hypothesised that these alterations may be specific to a subgroup of psychosis patients, examination of the distribution of immune markers does not support this [170]. Meta-analytic evidence also indicates that treatment with antipsychotic medication is associated with a reduction in with IL-6, whereas IL-12 and TNF-α remain raised despite treatment [172], suggesting that cytokines may function as both state and trait markers of psychosis. Moreover, elevations in IL-6 and IFN-γ have been associated with poorer responses to antipsychotic treatment [173]. Studies of medication-naïve psychosis patients have also observed raised B cells and reduced T cells with a raised CD4:CD8 sub-set ratio, which has been shown to normalise with antipsychotic treatment [171, 174]. Furthermore, some (but not all) studies have shown that CRP levels are associated with symptom severity and acute-phase illness among patients with schizophrenia [163]. With regards to how these immune markers might relate to psychosocial stress, studies observing elevated cytokine levels in FEP patients have also found that stress measures (life events and childhood trauma) are associated with raised cytokine levels, including TNF-α, IL-1β, IFN-γ, IL-6 [175] and TGF-β [176] in patients and/or controls.

#### 4.2.3 High Risk and Transition to First Episode Psychosis

In contrast to findings in patients with established psychosis, meta-analyses have provided no convincing evidence of abnormal immuno-inflammatory process in high-risk individuals. A recent meta-analysis (Table **2**) of 12 immuno-inflammatory markers observed no significant differences in any marker when all high-risk groups (CHR-P and FHR) were compared with controls [177]; although subgroup analyses showed significantly increased IL-6 levels in the CHR-P group only (g = 0.33, 95% CI: 0.06 to 0.60).

Some promising findings have been obtained using multivariable prediction models. For example, a study utilising data from small subset of the NAPLS-2 cohort (including 72 individuals at CHR) found that cortisol and several cytokines were among the 15 markers (of 117 tested) that best distinguished individuals at CHR-P who transitioned to psychosis from those who did not and healthy controls [178]. More recently, proteomic analysis of the ALSPAC and EU-GEI cohorts combined with clinical data was used to derive a model with an area under the curve (AUC) of 0.95 [179], where complement and coagulation proteins were highly implicated in the proteomic differences. Conversely, autoantibodies against the NMDA receptor (detectable in a small proportion of the EU-GEI cohort, but not more commonly than controls (8.3% *vs*. 5.2%; OR = 1.50, 95% CI: 0.58-3.90) were not associated with transition to psychosis [180].

### 4.3 Brain Structure

#### 4.3.1 Influence of HPA and Immuno-inflammatory Systems on the Brain

As described in Section 4.1, cortisol exerts its functions in the CNS by binding to MRs and GRs. Both receptors are highly expressed in the human hippocampus, although GRs are also distributed in other regions such as the amygdala and prefrontal cortex. These regions influence glucocorticoid release and behavioural responses to stress [181]: the hippocampus and prefrontal cortex inhibit HPA axis activity and participate in the regulation of the HPA axis by means of glucocorticoid feedback inhibition, whilst the amygdala can influence HPA axis when activated by emotional and physiological stressors.

These brain regions also determine how the brain adapts to stress, with the amygdala and hippocampus involved in emotional and contextual aspects, respectively, whilst the prefrontal cortex is involved in planning and control. There is some evidence to suggest that chronic stress induces structural changes in brain regions. Whilst low-stress or low-cortisol concentrations have particular neurotrophic effects, prolonged high stress (in dose or time) is thought to induce neurotoxicity by different mechanisms such as the glutamate cascade, the inhibition of glucose transport, and reducing brain-derived neurotrophic factor (BDNF) expression [182]. Chronic exposure to stress hormones has been shown to adversely affect the brain structures involved in cognition and psychiatric disorders, where the timing and duration of the exposure is a crucial factor [183, 184]. Specific brain areas may be more sensitive to the effects of stress hormones during periods when they are undergoing development; for example, the hippocampus (which undergoes significant development during the first two years of life) may be more vulnerable to early-life stressors [183] whereas stress exposure during late childhood and adolescence might lead to changes in amygdala volume and the frontal cortex, respectively (as these regions continue to develop in these developmental periods).

While the brain was long considered to be an immune-privileged site (due to the blood-brain barrier), evidence of complex interactions between the brain and the immune system has emerged. In addition to vagal afferents (crucial for bidirectional communication in the gut-brain axis) and circumventricular organs (small ventricular structures which lack a blood-brain barrier), there is evidence that meningeal lymphatic vessels are rich in immune cells and can play a role in regulating neuronal networks [185]. Moreover, brain-resident glial cells can transduce and integrate multiple signals, including from stress and systemic inflammation, leading to changes in neurobiology both structurally and functionally,for example, by synaptic pruning [186]. These findings support the notion that immuno-inflammatory processes can contribute to neuroanatomical and neurofunctional changes relevant to diathesis-stress models of psychosis.

#### 4.3.2 The Impact of Early Life Stress on the Brain

Stressors experienced during early life, including those occurring before (prenatal stress) and after birth (postnatal stress, childhood, and adolescence), have been shown to impact on brain structure and function. Converging evidence indicates that prenatal stress influences the brain and HPA axis by means of programming effects [187]. For example, prenatal stress has been associated with decreased functional connectivity and increased structural connectivity between the amygdala and the medial prefrontal cortex [188]. Moreover, foetal exposure to elevated placental CRH levels has been related to increased cortical thinning of temporal and frontal regions [189], an effect that may be more pronounced in females. With regard to postnatal stressors, childhood maltreatment has been found to correlate with structural and functional brain changes. Meta-analyses (Table **3**) have shown reduced grey matter in the right dorsolateral prefrontal cortex and right hippocampus among adults with a history of childhood trauma [190], as well as significantly increased activation in the left superior frontal gyrus and left middle temporal gyrus, and decreased activation in the left superior parietal lobule and the left hippocampus [191]. Studies also suggest that there might be sex-differences in the sensitivity of brain regions to childhood maltreatment [192], as early-life stress has been associated with lower hippocampus-subgenual cingulate resting-state functional brain connectivity (rs-FC) in both adolescent females and males but lower amygdala-subgenual cingulate rs-FC in females.

With regards to psychosis high-risk populations, a recent study reported that childhood sexual abuse was associated with reduced cortical thickness in bilateral middle temporal gyri and the left superior frontal gyrus in CHR-P individuals, whilst physical abuse was associated with increased cortical thickness in the right middle frontal gyrus [193]. Other studies exploring adversity in CHR-P youth have reported volume losses in the cortex and hippocampus in people with a history of deprivation but not threat [194]. Moreover, severity of childhood maltreatment has been correlated with resting cerebral blood flow (rCBF) in the bilateral hippocampus and negatively associated with rCBF in the left prefrontal cortex in CHR-P individuals [195].

#### 4.3.3 Stress Biomarkers and Brain Structure in Psychosis

Stress biomarkers may contribute to some of the neuroanatomical features associated with psychosis. An early longitudinal study observed that diurnal cortisol levels were inversely correlated with left hippocampal volume in patients with FEP [196]. In a further study from this group, childhood maltreatment and stressful life events were both found to contribute to a reduction in BDNF mRNA levels in FEP patients, possibly through a stress-induced increase in IL-6 expression [197]. Other studies have reported that a decreased CAR is associated with reduced hippocampal volumes in male FEP patients [198] and male CHR-P individuals [199]. With regards to immuno-inflammatory markers, a recent review of 14 publications concluded that there was reasonably consistent evidence that increased peripheral inflammation is associated with smaller hippocampal volume and reduced cortical thickness among patients with psychosis [200]. Psychosocial stress may also influence brain function: a recent fMRI study observed that the relationship between cortisol reactivity (the slope between pre- and post-imaging salivary concentrations) and brain activation in the bilateral temporo-parieto-insular junctions, right middle cingulum, right pre/postcentral gyri, left cerebellum and right lingual gyrus during an emotional face-matching task was moderated by the severity of childhood maltreatment and these effects were different in patients with schizophrenia and healthy controls [201]. Moreover, data from a large sample of CHR-P individuals observed that higher levels of proinflammatory cytokines at baseline were associated with greater prefrontal cortical thinning in the total sample and that this association was even more pronounced among those who transitioned to psychosis within two-years [202]. Thus, models incorporating markers of immuno-inflammatory processes may have utility in predicting neuroanatomical changes as well as symptom progression.

### 4.4 Cognition

#### 4.4.1 Glucocorticoids and Cognition in Non-psychiatric Populations

Studies of patients with endocrine disorders (*e.g*., Cushing’s syndrome) and healthy individuals indicate that chronic stress and excess glucocorticoids are associated with cognitive impairments. For example, patients with Cushing’s syndrome, where there is an excess of glucocorticoids (due to exogenous or endogenous factors), show deficits in verbal, visual, and working memory domains [203, 204], which improve following successful treatment. Some brain changes associated with Cushing’s syndrome, such as hippocampal atrophy, are also reversible with treatment [205, 206], although longer periods of glucocorticoid excess are associated lower likelihood of improvement [207]. Consistent with these findings, a longitudinal study of healthy subjects observed that individuals characterised by an increasing/high pattern of cortisol secretion (*i.e*., year-to-year increases in cortisol with currently high levels) showed significant impairments in declarative memory and selective attention compared to individuals with increasing/moderate or decreasing/moderate cortisol levels [208]. These results suggest that cognitive deficits linked to glucocorticoid excess are not specific to hippocampal function, since they also target the process of selective attention sustained by the frontal lobes. Several cohort studies have investigated whether cortisol may accelerate cognitive decline in humans. In a meta-analysis including 19 prospective studies (Table **4** [209]), higher morning cortisol was considered a risk factor for cognitive decline in mild cognitive impairment or mild Alzheimer’s Disease patients, although the results in cognitively healthy adults were inconsistent.

#### 4.4.2 Glucocorticoids and Cognition in Psychosis

Cognitive abnormalities are considered a core feature of schizophrenia and related psychoses [210]. These abnormalities are present (albeit to a lesser degree) in CHR-P populations before the psychosis onset [211], although these deficits appear to be attributable to those who subsequently transition to psychosis [212]. The neurotoxic hypothesis proposes that stress-induced HPA axis hyperactivity leads to an increase in cortisol which has neurotoxic effects on specific brain regions that express high levels of GRs (hippocampus and prefrontal cortex), thereby leading to cognitive impairments in domains attributed to these regions (*e.g*., memory and executive function) [10]. In support of this hypothesis, elevations in afternoon cortisol levels have been associated with poorer verbal memory in males with first-episode schizophrenia [213] whilst higher hair cortisol concentrations have been associated poorer working memory in psychosis patients and healthy controls, independent of diagnosis [214]. In contrast, a lower CAR has been related to a poorer processing speed and verbal memory performance in FEP patients [215]. Consistent with these findings, a study of children at elevated risk for psychosis (children at FHR and those presenting PLEs and other antecedents) observed that more pronounced HPA axis abnormalities (higher diurnal cortisol levels and reduced CAR) were associated with poorer verbal memory and executive functioning in high-risk groups, but this effect was not present in typically-developing children [216]. As these findings go against the neurotoxic hypothesis, the authors concluded that altered neurodevelopmental trajectories may have contributed to structural and functional abnormalities in regions that mediate both cognitive and HPA axis function. However, in a further study supporting the neurotoxic hypothesis, sex differences in the relationship between the CAR and verbal memory and processing speed were observed (a higher CAR was associated with poorer cognitive outcomes in female patients), as well as with the diurnal cortisol slope and spatial working memory (a flattened cortisol slope was associated with poorer working memory in female patients) [217]. Immuno-inflammatory processes might plausibly contribute to the cognitive impairments that characterise individuals with psychosis [218]; however, findings have been mixed (Table **4**). A recent systematic review observed that CRP levels were inconsistently associated with cognitive impairment in patients with psychosis [163], similarly, a meta-analysis that included studies of patients with psychotic and mood disorders found limited evidence to suggest that immune markers were associated with cognitive performance in the combined patient analysis or when stratified by diagnostic subgroup [219]. In contrast, a subsequent meta-analysis of studies examining patients with schizophrenia concluded that cognitive deficits were associated with elevations in pro-inflammatory markers in this population [220]. Given the inconsistency of findings across studies, further research investigating the interplay between early-life stress, stress biomarkers, and cognitive outcome is an important area for future research.

### 4.5 Gut Microbiome

The gut microbiome is a complex and dynamic ecosystem of microorganisms present in the intestinal tract [221]. Humans host around 500-1000 bacterial species, with a total gene set 150 times larger than the human genome [222, 223]. Gut bacteria contribute to many of the host’s physiological functions, including nutrient metabolism, immunomodulation, and permeability of biological barriers [223]; in turn, the host sends signals to the gut and modulates the composition and functionality of gut bacteria [221]. A consistent body of evidence now suggests that the gut microbiome can affect brain chemistry and development, including key nodes of the physiological stress system [224-226]; however, most of this work is preclinical, with few studies in humans.

#### 4.5.1 Effects of the Stress System on the Gut Microbiome

Converging evidence indicates that several psychosis risk factors can modify the composition and functionality of the gut microbiome [227], which in turn, can affect specific gut-brain pathways believed to contribute to the pathophysiology of psychosis. In prenatal life, a recent study in humans showed that infants from mothers who exhibited high levels of self-reported stress and salivary cortisol during pregnancy had lower levels of Lactobacilli and Bifidobacteria in the gut [228]. Similar findings were obtained in newborn pups of pregnant mice exposed to severe stress during pregnancy [229] where gut microbiome shifts were accompanied by modifications in the levels of metabolites and essential amino acids in the hippocampus and thalamus of the pups. Similarly, adverse childhood experiences (*e.g*., parent-child dysfunctions, lower socioeconomic status, and childhood trauma) have also been associated with altered gut microbiome composition in humans [230, 231]. Consistent with these observations, early maternal separation in non-human animals induces significant shifts in the gut microbiome of the offspring [232, 233], which is accompanied by increased peripheral inflammation (IFN-γ and IL-6) and altered central neurotransmission [233]. With regards to other psychosis-relevant risk factors, migration [234] and urbanisation [235] have both been associated with reduced gut microbiome diversity.

#### 4.5.2 Effects of the Gut Microbiome on the Stress System

The most convincing evidence that the gut microbiome influences the stress system comes from studies of mice never exposed to bacteria (germ-free mice, GFM) and antibiotic-depleted mice. Studies of GFM indicate that these animals exhibit abnormal activation of the HPA axis (increases in stress hormones and locomotor activity) when exposed to stressors [236]. However, as GFM have abnormal brain development and are often raised in social isolation (germ-free environment), some authors questioned the validity of these findings [236]. This criticism was recently addressed in studies using co-caged antibiotic-depleted mice, which have physiological brain development and avoid the germ-free environment bias. Consistent with the findings from GFM, antibiotic-depleted mice also showed a heightened activation of the HPA axis in response to a range of different stressors [237, 238]. These studies also demonstrate that the lack of bacteria induced by antibiotic depletion in mice is associated with hippocampal atrophy and reduced neuroplasticity [238], features which have been consistently associated with both heightened stress exposure and psychosis [239]. Importantly, these central detrimental effects could be prevented by modulating the gut microbiome with different interventions (*e.g*., probiotics and faecal transfer from non-antibiotic-depleted mice) [240]. Such findings are in line with those obtained in humans, where probiotics and prebiotics can reduce stress perception and cortisol release in otherwise healthy individuals [241, 242].

Animal studies indicate that the efficacy of gut microbiome-based interventions is dependent on developmental stage, with earlier interventions resulting in more consistent and long-lasting neuroprotective effects [240]. This is not surprising, given that the timeline of the development of the mammalian stress system coincides with the acquisition and consolidation of the gut microbiome [225]. In humans, the circadian rhythm of cortisol release consolidates in the early postnatal period, approximately six months after birth [243], this period also represents a key window for the colonisation of the gut by bacteria. Evidence suggests that the gut is sterile at birth [244], although recent work shows that initial bacterial colonisation might occur as early as 10 weeks after conception when the foetus begins to swallow amniotic fluid [245]. Immediately after birth, the gut of the infant is colonised by bacteria coming either from the mother’s vagina (in case of vaginal delivery) or from the mother’s/carer’s skin (in case of C-section) [245]. Once feeding commences, additional bacteria can be acquired from breast milk [245]. These initial steps of bacterial colonisation introduce a high degree of interindividual variability in gut microbiome composition. For example, formula-fed and C-section born infants have a lower abundance of *Bifidobacteria* and *Bacteroidetes* (essential for the digestion of human milk oligosaccharides) and greater levels of Proteobacterial groups (known to contain pathogens) [246]. These differences might result in reduced absorption of essential nutrients and increased activation of the immune system, both having important effects on the brain and specific HPA axis components (namely, cytokine-induced release of CRH from the hypothalamus) [247], especially during this critical developmental period. Indeed, C-section and formula-feeding are associated with an altered stress response that persists into adulthood and with an increased risk of immunological disorders [248, 249], but the link with psychotic disorders is more tenuous [250]. It is plausible that these associations might be mediated by alterations of the gut microbiome, although this remains to be tested. Other critical periods for the development of the HPA axis are childhood and early adolescence [243], where the gut microbiome is reaching its final and more stable composition [221].

#### 4.5.3 The Gut Microbiome in Psychotic Disorders

Accumulated evidence suggests that reduced microbiome diversity may contribute to a number of detrimental health outcomes, including psychiatric disorders, although meta-analyses (Table **5**) have yielded variable findings depending on the microbiome measure and psychiatric population examined [251, 252]. Nevertheless, studies have shown that gut microbiome composition is altered in psychosis patients [253-255] and individuals at CHR-P [256] when compared to controls, independent of medication [251, 253]. Moreover, gut microbiome samples obtained from medication-naive patients can cause brain and behavioural abnormalities in recipient mice that resemble those of schizophrenia [251]. Of translational relevance, recent studies suggest that gut microbiome features can be used to predict treatment response [257] and functional deterioration [255] in patients with psychosis.

Multiple mechanisms have been hypothesised to explain these findings. For example, the gut microbiome is a key regulator of immune system development [258]; shifts in the gut microbiome have been associated with modifications of the activity of the complement system and increased production and release of cytokines and autoantibodies with anti-glutamatergic action [258-260]. This enhanced immunological activation is a well-established feature of novel psychopathological models of psychotic disorders [165]. Other mechanisms include the regulation by bacteria of the circulating levels of kynurenate, an NMDA receptor antagonist [261]; the synthesis of short-chain fatty-acids (acetate, butyrate, propionate) that modulate the permeability of the blood brain barrier [262]; the synthesis of endocannabinoids and other neurotransmitters [263]; and the regulation of the activity of the enteric nervous system and the stress system [264]. However, studies in clinical samples are needed to test these hypothesised mechanisms.

## 5 IMPLICATIONS FOR PREVENTION AND TREATMENT

We now turn our attention to the real-world implications, that is, how might our understanding of the stress neurobiological mechanisms be used to improve outcomes for those at risk for psychosis. However, a major limitation is that work in this field has thus far focused on individuals with established psychosis.

### 5.1 Antipsychotic Medication

There is some evidence to suggest that antipsychotic medications may influence the physiological systems described in the preceding sections, and that these actions may partially explain their therapeutic effects. With regards to the HPA axis, an observational study of FEP patients found that those with < 2 weeks of antipsychotic treatment were characterised by significantly higher diurnal cortisol levels when compared to patients with > 2 weeks of treatment and healthy controls [265]. Experimental studies have confirmed this pattern, for example, a double-blind randomised controlled trial (RCT) examined the effect of antipsychotic administration *vs*. placebo in healthy controls (n = 11) and found that quetiapine and olanzapine significantly reduced levels of plasma cortisol and ACTH levels [266]. A larger double-blind RCT of patients with treatment-resistant schizophrenia (n = 78) observed that 12-weeks treatment with risperidone or haloperidol both resulted in a decrease in serum cortisol levels and that cortisol levels at follow-up were associated with negative symptoms [267]. Similar findings have been observed for cytokines; indeed, this same trial observed that risperidone and haloperidol were both associated with a reduction in IL-2 (but not IL-6) levels and that pre-treatment IL-2 levels were associated with positive symptoms. Moreover, a meta-analysis of eight observational studies (Table **6**) observed that IL-2 and IL-6 levels were significantly decreased (with moderate effect sizes) in FEP patients following four weeks of antipsychotic treatment [268]. With regards to the gut-microbiome, there appears to be a reciprocal relationship, in that antipsychotic medications directly affect the composition of the gut-microbiome, and in turn, microbiota influence the absorption (and hence efficacy) of antipsychotics [269]. Specifically, a large *in vitro* study that investigated more than 1,000 non-antibiotic medications, found that antipsychotic medications were among the three classes of medications that were found to inhibit gut bacteria to a greater degree than other medications [270]. Moreover, as this study also found that gut microbes targeted by different antipsychotics were more similar than would be expected based on the structural similarity of these medications, the authors concluded that these bactericidal effects may be part of the therapeutic action rather than simply a side effect.

### 5.2 Treatments Targeting the HPA Axis

Evidence of cortisol abnormalities in patients with psychosis has generated interest in assessing the therapeutic benefit of antiglucocorticoid treatments (either as a sole or adjunctive treatment) in this population. These treatments typically inhibit cortisol synthesis (*e.g*., metyrapone, aminoglutethirnide, ketoconazole) or act as GR antagonists (*e.g*., mifepristone) thereby suppressing cortisol secretion [271]. A Cochrane review of RCTs found insufficient evidence to indicate that antiglucocorticoid medications improved psychotic symptoms (either positive or negative) when administered as an adjunctive treatment [272]. However, the largest of these studies (n = 221) examined outcomes among patients with psychotic depression after seven days of mifepristone treatment (followed by treatment-as-usual) and reported that these patients were significantly more likely to achieve treatment response (defined as a 30% reduction in global symptoms) than those receiving placebo [273]. As noted in this review, study quality was highly variable (many studies had small samples and short follow-up periods), thereby limiting the ability to draw conclusions regarding the potential utility of targeting the HPA axis system. It is possible that any potential benefits of antiglucocorticoid medications might be restricted to a subset of individuals with HPA axis abnormalities at treatment commencement. Indeed, a recent meta-analysis (Table **6**) examining the efficacy of antiglucocorticoid treatment in mood disorders reported that patients who responded to treatment with cortisol synthesis inhibitors had significantly higher peripheral cortisol levels at baseline than non-responders [274], suggesting that only those with HPA axis abnormalities at baseline might benefit from treatment.

### 5.3 Therapies Targeted at Immune and Inflammatory Abnormalities

Since there are extensive therapeutics targeting inflammatory mediators that could be repurposed, there has been an interest in evaluating whether this may have a treatment role in psychosis. Early studies investigated broad anti-inflammatory treatments such as aspirin or non-steroidal anti-inflammatory drugs, observing modest effects ([275, 276], Table **6**). More recently, specific therapies have been tested; however, a small placebo-controlled RCT of tocilizumab, a monoclonal antibody against IL-6, observed no effect on symptom severity at 12 weeks in patients with schizophrenia [277]. The authors proposed that future trials might consider enriching samples with individuals presenting inflammatory biomarker abnormalities at recruitment, targeting earlier in the illness, and treating those with more severe symptoms. A further perspective is to consider trans-diagnostic subsyndromal aspects of psychosis such as anhedonia, that are associated with differential cytokine signatures and investigate the effects of anti-inflammatory medications on these specific symptoms in trials across psychosis and mood disorders [278, 279].

### 5.4 Treatments Targeting the Gut-microbiome

Treatments targeting the gut-microbiome include probiotics (live microorganisms that confer health benefits to the host when administered in sufficient quantities) and prebiotics (substrates that benefit the host by selectively stimulating growth and/or activity of gut microorganisms) [280]. There has been substantial interest in evaluating the effect of these nutritional supplements in patients with psychiatric disorders, with an initial review of the literature (including studies published between 2014-2019) concluding that there was some evidence to suggest that probiotics may improve depression, but not anxiety or schizophrenia [281]. Similarly, a subsequent meta-analysis (Table **6**) found that across 28 RCTs evaluating add-on strategies targeting the gut-microbiome (of which 21 examined antibiotics, with only three investigating pre-and/or probiotics) there was no evidence to suggest that augmentation was significantly superior to placebo in improving symptom severity in patients with schizophrenia [282].

## 6 DISCUSSION

We have described a network of integrated systems that are relevant to understanding how psychosocial stressors contribute to the risk of psychosis. Our review shows that whilst there is reasonably consistent evidence that individuals with established illness are characterised by higher levels of traditional stress biomarkers (namely cortisol and cytokines), studies of high-risk groups have yielded highly variable findings. Moreover, treatments targeting the HPA axis, immuno-inflammatory processes, and the gut-microbiome have yet to be convincingly shown to improve treatment outcomes among those with established psychosis, although some of the beneficial effects of antipsychotics may be through these systems.

Whilst these findings are somewhat discouraging, the lack of consistency is unsurprising for several reasons. First, the mode of onset, treatment course, and long-term prognosis for psychotic disorders is highly variable [1], thus, it is implausible that a single underlying biological mechanism could be associated with illness onset, symptom expression, and treatment response. Indeed, there is evidence to suggest that psychotic disorder subtypes are associated with distinct neurobiological features: for example, shared and distinct brain regions appear to underlie the abnormalities of sustained attention that characterise both affective and non-affective psychotic disorders [283]. Moreover, there is sexual-dimorphism (both structural and functional) in the brain systems associated with psychosis, which may contribute to differences in treatment response across males and females [284]. Heterogeneity is even more pronounced in high-risk samples, with meta-analyses including help-seeking CHR-P individuals presenting symptoms consistent with the prodrome, through to members of the general population who present PLEs that are often benign and not distressing. Even within the CHR-P group, there is considerable variation, where individuals presenting with BLIPS (which form only a small proportion of CHR-P individuals) have the highest liability for transition [38]. Thus, the probability of finding any mean difference in biomarkers in high-risk groups relative to controls is further reduced. Second, given the wealth of psychosocial stressors that increase psychosis risk, it is unsurprising that studies examining the correspondence between stress exposure and neurobiological markers have failed to show evidence of an association [157]. Such studies typically measure one, or a limited set, of psychosocial stressors and correlate these with biomarker levels. Yet, even if we assume that psychosocial stress contributes to illness onset among all individuals with psychosis (which is highly unlikely), as there is no stressor experience that is common to all individuals with psychosis, the ability to detect associations with physiological parameters is greatly diminished. Adding to this complexity, these stressors operate throughout the lifespan, ranging from events in early childhood which may set the scene for emerging psychopathology through to ‘triggering’ life events occurring proximal to illness onset. The fact that these stressors are commonly assessed retrospectively, with hypothesised biomarkers measured many years later, further reduces the ability to detect associations. Finally, the neurobiological systems that are relevant to our understanding of the impact of psychosocial stress extend far beyond the HPA axis. As noted, the HPA axis has reciprocal relationships with immuno-inflammatory processes and the gut-microbiome, and changes within these systems can influence brain structure and function. However, these neurobiological mechanisms are often examined in isolation, using a single biomarker and the external (*e.g*., environment, diet, infections, substance use, and medication) and internal factors (*e.g*., somatic disorders, biological sex, age) that influence these mechanisms are scarcely measured [10]. As such, our ability to identify physiological correlates of psychosocial stressors, even where these are robustly associated with illness onset in a high proportion of individuals, is limited.

### 6.1 Moving the Translational Field Forward

**Fig. (1):**
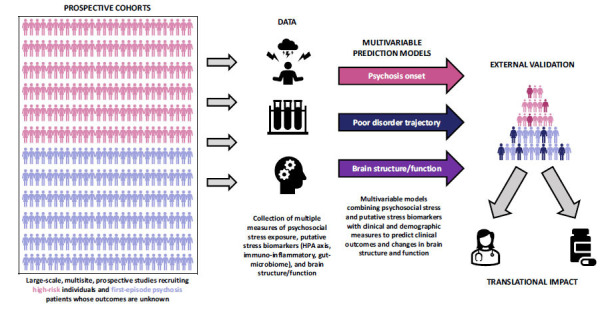
Proposed strategy for moving the translational field forward.

As we argue above, studies investigating isolated markers of single neurobiological systems, in small samples of FEP or high-risk individuals will continue to generate conflicting, and sometimes, spurious findings. We propose that a more useful strategy (Fig. **1**) may be to ensure that large-scale, multisite studies recruiting high-risk and FEP participants routinely collect data on stress exposure and a range of samples (blood, saliva, and stool) that can be used to assess multiple, putative markers of stress neurobiology (*i.e*., HPA axis, immuno-inflammatory, and gut-microbiome). In this way, we can determine the extent to which these data can be used alongside precision psychiatry methods to detect at-risk individuals, predict illness trajectories and psychosis onset, and stratify individuals to enable targeted interventions. Precision psychiatry tools provide individualised risk estimates to inform clinical decisions and can incorporate data on stress to refine and improve the accuracy of their predictions. Multivariable models combining clinical and/or sociodemographic data with stress exposure [96, 285, 286] or stress biomarkers [152] have already been developed and appear to have promise. However, most of them are not replicated and fail the implementation test [287-289], thus lacking clinical relevance. Better representation of interactions between risk factors in a multivariable approach (*e.g*., combining information on momentary stress with trauma history and cortisol levels) is essential for future research, particularly when considering multiple measures of stress, which will be highly correlated. Preliminary work has been encouraging [290], for example using polyenvironmental risk scores [285, 286, 291] but large, generalisable samples are needed to develop and validate these tools. External validation studies, where independent datasets are used to replicate model performance, are rare in psychiatry [288] but are needed in order to provide evidence that precision psychiatry tools can perform well in multiple settings and are viable for use in real-world clinical care [292].

## CONCLUSION

We propose that among individuals who are at high-risk for psychosis, there will be a small proportion for whom dysregulation within neurobiological stress systems is present, and within this group, some for whom this dysregulation is relevant to illness onset and course (rather than epiphenomenal). The next step is to develop methods to systematically identify these individuals and determine whether targeted interventions (*e.g*., supplementation with anti-inflammatory agents or gut-microbiome interventions) might be beneficial. The establishment of several international consortiums throughout the world will enable the collection of biomarkers to a much larger scale than we have seen previously. These efforts are needed to determine the translational utility of stress biomarkers in vulnerable populations, as evidence to support this is currently lacking.

## Figures and Tables

**Table 1:** Meta-analyses examining hypothalamic-pituitary-adrenal (HPA) axis markers in established psychosis and high-risk populations.

**Author**	**Population**	**Measure**	**Findings**
Berger *et al.* (2016)	Schizophrenia, FEP, CHR-P	Cortisol awakening response	Pooled analysis of all patient groups (schizophrenia, FEP, CHR-P) showed significantly lower CAR in patients relative to healthy controls (k = 11, g = -0.426 (95% CI: -0.585 to -0.267) *p* < 0.001). However, subgroup analyses indicated no differences in the CAR between CHR-P individuals and controls (k = 3, g = -0.170 (95% CI: -0.435 to 0.095) *p* = 0.207).
Ciufolini *et al.* (2014)	Schizophrenia	Cortisol response to psychosocial stressor tasks	Compared to controls, individuals with schizophrenia demonstrated significantly lower cortisol levels during the anticipatory phase of the psychosocial stressor task (k = 3, g = −0.39, (95% CI: −0.74 to −0.03), *p* = 0.032) and in response to the stressor (k = 3, g = −0.66, (95% CI: −1.20 to −0.12) *p* = 0.016); however, no significant differences in cortisol levels were observed during the recovery phase (k = 3, g = −0.34 (95% CI: −1.03 to 0.35) *p* = 0.33).
Chaumette *et al.* (2016)	FEP, CHR-P	Basal salivary cortisol	Basal salivary cortisol observed to be significantly increased in CHR-P individuals relative to healthy controls (k = 8, g = 0.59 (95% CI not reported) *p* = 0.004). Subgroup analysis showed no differences between CHR-P individuals who subsequently transitioned and those who did not (k = 4, g = not reported (95% CI not reported) *p* = 0.90). No significant differences in basal salivary cortisol in comparisons between FEP patients and healthy controls (k = 6, g not reported (95% CI not reported), *p* = 0.56).
Cullen *et al.* (2020)	Schizophrenia, FEP, CHR-P, FHR, SPD, PLEs	Stressor-cortisol concordance	When pooled across all populations (established psychosis, high-risk, healthy controls) only a weak, positive association observed between naturally occurring stressors and cortisol (k = 134, *r* = 0.05 (95% CI: -0.00 to 0.10) *p* = 0.059). Meta-regression analyses showed no significant effect of either established psychosis status (*r* = 0.01 (95% CI: -0.01 to 0.16) *p* = 0.838) or high-risk status (*r* = 0.02 (95% CI: -0.05 to 0.10), *p* = 0.477) on effect sizes, indicating that the degree of concordance in these groups did not differ from healthy controls.
Girshkin *et al.* (2014)	Schizophrenia	Morning cortisol	Morning cortisol levels significantly increased in schizophrenia patients relative to controls (k = 44, g = 0.387, (95% CI: 0.154 to 0.619) *p* = 0.001). Sub-analyses restricted to patients free from antipsychotic medication showed more pronounced increases of morning cortisol relative to controls (k = 16, g = 0.468 (95% CI: 0.242 to 0.693) *p* < 0.001).
Hubbard & Miller (2019)	First-episode psychosis	Blood cortisol	Blood cortisol levels significantly increased in FEP relative to controls (k = 27, SMD = 0.37 (95% CI: 0.16 to 0.57) *p* < 0.001), with larger effect sizes when restricted to antipsychotic naïve samples (k = 18, SMD = 0.46 (95% CI 0.20: to 0.73) *p* = 0.001).
Misiak *et al.* (2021a)	FEP, CHR-P, FHR,	Blood and salivary cortisol	Compared to healthy controls, FEP group showed significantly higher levels of unstimulated blood cortisol levels (k=22, g = 0.48, (95%CI = 0.25 to 0.70) *p* < 0.001), a lower CAR (k = 4, g = -0.40 (95% CI = -0.68 to -0.12) *p* = 0.006) but no differences in unstimulated salivary cortisol levels (k = 7, g = 0.09 (95% CI: -0.09 to 0.26) *p* = 0.331). In contrast, no differences between high-risk individuals and healthy controls were observed for unstimulated blood cortisol (k = 7, g = 0.39 (95% CI: - 0.42 to 1.21) *p* = 0.342), unstimulated salivary cortisol (k = 10, g = 0.15 (95% CI: -0.01 to 0.31) *p* = 0.062), or the CAR (k = 5, g = -0.13 (95% CI: -0.44 to 0.19) *p* = 0.433). Unstimulated salivary cortisol levels did not differ between CHR-P converters and non-converters (k = 4, g = -0.13 (95% CI: -0.50 to 0.24) *p* = 0.500.
Nordholm *et al.* (2013)	Schizophrenia, FEP, CHR-P, SPD,	Pituitary volume	No significant differences in pituitary volume observed in comparisons between all high-risk individuals (CHR-P and SPD) and healthy controls (k = 3, SMD = 0.25 (95% CI: -0.17 to 0.66), *p* = 0.24) or when CHR-P individuals who transitioned to psychosis were compared with healthy controls (k = 2, SMD = 0.37 (95% CI: 0.00 to 0.75) *p* = 0.05). Similarly, no significant differences in pituitary volume observed when all patients with established psychosis (schizophrenia and FEP) were compared with controls (k = 9, SMD = 0.10 (95% CI: -0.20 to 0.40) *p* = 0.52) or when restricted to patients with FEP (n = 4, SMD = 0.39 (95% CI: -0.05 to 0.84) *p* = 0.09).
Saunders *et al.* (2019)	CHR-P, FHR, SPD, PLEs	Pituitary volume	Significant increase in pituitary volume observed in the primary analysis comparing all high-risk groups (CHR-P, FHR, SPD, PLEs) and healthy controls (k = 11, g = 0.16 (95% CI: 0.00 to 0.32) *p* = 0.04). Pituitary volumes larger among high-risk individuals who later transitioned to psychosis when compared to controls (k = 4, g = 0.55 (95% CI: 0.06 to 1.04), *p* = 0.028). No significant differences observed in individual subgroup analyses comparing healthy controls with CHR-P individuals (k = 4, g = 0.17 (95% CI: -0.12 to 0.45) *p* = 0.25), or FHR groups (k = 4, g = 0.20 (95% CI. -0.03 to 0.43) *p* = 0.09).
Zorn *et al.* (2017)	Schizophrenia	Cortisol response to psychosocial stressor tasks	Individuals with schizophrenia showed diminished increase (AUCi) in cortisol levels (k = 4, SMD = -0.594 (95% CI: −1.150 to −0.037) *p* = 0.04) and lower overall cortisol levels (AUCg) during the stressor task (k = 4, SMD = -0.543 (95% CI: −0.968 to −0.118) *p* = 0.01) relative to healthy controls.

**Abbreviations:** FEP: first-episode psychosis; CHR-P: clinical high-risk; FHR: familial high-risk; SPD: schizotypal personality disorder; PLEs: psychotic-like experiences; CAR: cortisol awakening response; k: number of effect sizes included in analysis; g: Hedges g effect size; SMD: standardised mean difference; *r:* correlation coefficient; CI: confidence interval; AUCi: area under the curve with respect to increase; AUCg: area under the curve with respect to ground.

**Table 2:** Meta-analyses examining immuno-inflammatory markers in established psychosis and high-risk populations.

**Author**	**Population**	**Measure**	**Findings**
Miller *et al.* (2011)	Schizophrenia and FEP	Cytokines measured in blood	In acutely relapsed patients compared with controls, IL-10 levels were significantly decreased (k = 2, g = -0.57 (95% CI: -0.98 to -0.17) *p* = 0.006), levels of IL-6 (k = 6, g = 0.96 (95% CI: 0.74 to 1.18) *p* < 0.001), IL-8 (k = 2, g = 0.59 (95% CI: 0.18 to 1.00) *p* = 0.004), TNF-α (k = 4, g = 0.73 (95% CI: 0.46 to 0.99) *p* = 0.001), IFN-γ (k = 2, g = 0.49 (95% CI: 0.18 to 0.80) *p* = 0.002), TGF-β (k = 2, g = 0.53 (95% CI: 0.26 to 0.79) *p* < 0.001), and IL-1RA (k = 2, g = 0.49 (95% CI: 0.07 to 0.90) *p* = 0.02) were significantly increased, whilst IL-2 (k = 2, g = -0.26 (95% CI: -0.60 to 0.08) *p* = 0.13) and sIL-2R (k = 2, g = 0.27 (95% CI: -0.13 to 0.68) *p* = 0.19) were not significantly different compared to controls. In medication naive FEP patients *vs*. controls, IL-1β (k = 3, g = 0.60 (95% CI: 0.37 to 0.84) *p* < 0.001), IL-6 (k = 4, g = 1.40 (95% CI: 1.14 to 1.65) *p* < 0.001), IL-12 (k = 2, g = 0.98 (95% CI: 0.61 to 1.35) *p* < 0.001), IFN-γ (k = 2, g = 0.57 (95% CI: 0.24 to 0.90) *p* = 0.001), TNF-α (k = 4, g = 0.81 (95% CI: 0.61 to 1.01) *p* < 0.001), TGF-β (k = 2, g = 0.48 (95% CI: 0.22 to 0.74) *p* < 0.001), and sIL-2R (k = 3, g = 1.03 (95% CI: 0.55 to 1.52) *p* < 0.001) levels were significantly decreased relative to controls, with no differences in IL-2 (k = 4, g = -0.09 (95% CI: -0.32 to 0.14) *p* = 0.44).
Miller *et al.* (2013)	Schizophrenia and FEP	Lymphocytes	Relative to controls, acutely relapsed patients showed increased total WBC (k = 2, g = 0.54 (95% CI: 0.08 to 1.00) *p* = 0.02), CD4% (k = 5, g = 0.26 (95% CI: 0.02 to 0.50) *p* = 0.04), and CD56% (k = 2, g = 0.83 (95% CI: 0.41 to 1.25) *p* = <0.01), but no differences in total lymphocyte count (k = 6, g = -0.04 (95% CI: -0.29 to 0.20) *p* = 0.72), CD3 (k = 5, g = 0.04 (95% CI: -0.19 to 0.27) *p* = 0.74), CD3% (k = 5, g = -0.12 (95% CI: -0.36 to 0.11) *p* = 0.31), CD4 (k = 4, g = 0.05 (95% CI: -0.20 to 0.30) *p* = 0.70) CD8 (k = 4, g = -0.16 (95% CI: -0.40 to 0.09) *p* = 0.22), CD8% (k = 5, g = 0.07 (95% CI: -0.17 to 0.31) *p* = 0.57), CD4/CD8 (k = 6, g = 0.15 (95% CI: -0.07 to 0.36) *p* = 0.19), CD19 (k = 4, g = 0.17 (95% CI: -0.08 to 0.42) *p* = 0.18), CD19% (k = 3, g = 0.0 (95% CI: -0.32 to 0.33) *p* = 0.98), CD25% (k = 2, g = -0.05 (95% CI: -0.45 to 0.36) *p* = 0.81), or CD56 (k = 3, g = 0.26 (95% CI: -0.06 to 0.57) *p* = 0.11). Medication-naive FEP patients showed significantly increased total lymphocyte count (k = 2, g = 0.77 (95% CI: 0.25 to 1.29) *p* = <0.01), CD3 (k = 2, g = 0.72 (95% CI: 0.20 to 1.24) *p* = <0.01), CD4 (k = 2, g = 0.86 (95% CI: 0.33 to 1.38) *p* = <0.01), and CD4/CD8 (k = 2, g = 0.56 (95% CI: 0.04 to 1.07) *p* = 0.03) levels relative to controls, but no differences in CD3% (k = 3, g = -0.31 (95% CI: -0.63 to 0.00) *p* = 0.05), CD4% (k = 3, g = 0.02 (95% CI: -0.29 to 0.33) *p* = 0.90), CD8 (k = 2, g = 0.44 (95% CI: -0.08 to 0.95) *p* = 0.10), CD8% (k = 3, g = -0.03 (95% CI: -0.34 to 0.29) *p* = 0.88), or CD19 (k = 2, g = 0.30 (95% CI: -0.09 to 0.69) *p* = 0.13).
Misiak *et al.* (202b)	FHR, CHR-P	Immuno-inflammatory markers	In analyses including all available high-risk groups (CHR-P and FHR) no significant differences observed in levels of CRP (k = 9, g = 0.08 (95% CI: -0.12 to 0.28) *p* = 0.448), IL-1β (k = 4, g = 0.13 (95% CI: -0.13 to 0.40) *p* = 0.323), IL-4 (k = 2, g = 1.50 (95% CI: -0.52 to 3.51) *p* = 0.145), IL-5 (k = 2, g = -0.17 (95% CI: -0.51 to 0.18) *p* = 0.349), IL-6 (k = 12, g = 0.18 (95% CI: -0.02 to 0.39) *p* = 0.082), IL-8 (k = 4, g = 0.35 (95% CI: -0.23 to 0.93) *p* = 0.242), IL-10 (k = 3, g = 0.27 (95% CI: -0.16 to 0.70) *p* = 0.222), IFN-γ (k = 7, g = 0.08 (95% CI: -0.25 to 0.41) *p* = 0.625), TNF-α (k = 8, g = 0.10 (95% CI: -0.19 to 0.39) *p* = 0.506), IL-12p70 (k = 3, g = 0.17 (95% CI: -0.19 to 0.53) *p* = 0.363), MCP-1 (k = 3, g = 0.55 (95% CI: -0.43 to 1.53) *p* = 0.273), or eotaxin-1 (k = 2, g = 0.76 (95% CI: -0.47 to 2.00) *p* = 0.226) when compared with healthy controls. In subgroup analyses, significantly higher levels of IL-6 were observed in CHR-P individuals relative to controls (k = 7, g = 0.33 (95% CI: 0.06 to 0.60) *p* = 0.018).
Pillinger *et al.* (2019)	FEP	Immuno-inflammatory markers	Compared to healthy controls, antipsychotic naive FEP patients showed significantly higher levels of IFN-γ (k = 9, g = 0.32 (95% CI = 0.11–0.53) *p* = 0.003), IL-17 (k = 7, g = 0.48 (95% CI = 0.06– 0.89) *p* = 0.03), IL-6 (k = 15, g = 0.62 (95% CI = 0.32–0.92) *p* < .0001), TGF-β (k = 3, g = 0.53 (95% CI = 0.18–0.88) *p* = .003), and TNF-α (k = 11, g = 0.56 (95% CI = 0.22–0.90) *p* = 0.001), but no differences in CRP (k = 5, g = 0.66 (95% CI = −0.03 to 1.34) *p* = 0.06), total lymphocyte count (k = 3, g = 0.31 (95% CI = −0.13 to 0.76) *p* = 0.17), IL- 10 (k = 9, g = 0.24 (95% CI = −0.13 to 0.62) *p* = 0.20), IL-1β (k = 7, g = 0.49 (95% CI = −0.13 to 1.11) *p* = 0.12), IL-2 (k = 9, g = −0.07; (95% CI = −0.53 to 0.39) *p* = 0.77), IL-4 (k = 7, g = 0.23 (95% CI = −0.05 to 0.51) *p* = 0.10), IL-8 (k = 5, g = 0.04 (95% CI = −0.62 to 0.70) *p* = 0.90), or sIL-2R (k = 4, g = 2.66 (95% CI = −0.03 to 5.34) *p* = 0.05).

**Abbreviations:** CHR-P: clinical high-risk; FHR: familial high-risk; FEP: first-episode psychosis; k: number of effect sizes included in analysis; g: Hedges g effect size; SMD: standardised mean difference; CI: confidence interval; CRP: C-reactive protein; IL: interleukin; IFN: interferon; TNF: tumour necrosis factor; TGF: transforming growth factor; MCP-1: monocyte chemoattractant protein-1; WBC: white blood cell count.

**Table 3:** Meta-analyses examining associations between childhood trauma and neuroimaging measures in clinical and non-clinical populations.

**Author**	**Population**	**Measure**	**Findings**
Heany *et al.* (2018)	Adults (clinical and non-clinical)	fMRI responses to socioaffective cues and childhood trauma	Across all studies reporting positive associations (k = 9), CTQ scores positively associated with brain activation in the left superior frontal gyrus extending into the medial frontal gyrus. Across all studies reporting negative associations (k = 6), brain activation in a cluster within the left superior parietal lobule was negatively associated with CTQ scores.
Paquola *et al.* (2016)	Adults (clinical and non-clinical)	Grey matter volume and childhood trauma	In the analysis of hippocampal volume, across all study samples (including clinical and non-clinical populations: k = 17) childhood trauma was associated with a significant reduction in left (g = −0.642, *p* = 0.001), right (g = −0.616, *p* < 0.001) and combined hippocampal volume (g = −0.517, *p* < 0.001). When stratified by disorder group (healthy controls, PTSD, BPD, mood disorder), reductions were significantly greater in the BPD cohort relative to controls and this was the only group to show reductions in left, right, and combined hippocampal volume. For amygdala volume, across all studies (including clinical and non-clinical populations: k = 13) childhood trauma was associated with a significant reduction in left (g = −0.482, *p* = 0.006), right (g = −0.668, *p* = 0.002) and combined amygdala volume (g = −0.559, *p* < 0.001), effect sizes more pronounced when restricted to psychiatric cohorts for left (g = −0.926, *p* < 0.001), right (g = −1.234, *p* < 0.001) and combined amygdala volume (g = −1.020, *p* < 0.001) In the analysis of WBV derived from VBM, analysis of all samples (k = 19) indicated that childhood trauma was associated with significantly reduced grey matter in three clusters: right dorsolateral prefrontal cortex (SMD = −1.897, *p* < 0.001), right hippocampus (SMD = −1.718, *p* < 0.001), and right postcentral gyrus (SMD = −1.582, *p* = 0.002).

**Abbreviations:** fMRI: functional magnetic resonance imaging; CTQ: childhood trauma questionnaire; k: number of effect sizes included in analysis; PTSD: post-traumatic stress disorder; BPD: borderline personality disorder; g: Hedges g effect size; SMD: standardised mean difference; WBV: whole brain volume; VBM: voxel based morphometry.

**Table 4:** Meta-analyses examining associations of cortisol and immune markers with cognitive function in clinical populations.

**Author**	**Population**	**Measure**	**Findings**
Morrens *et al.* (2022)	Psychotic and mood disorders	Immune markers and cognitive dysfunction	In analysis including all psychotic and mood disorders patients, proinflammatory markers were negatively correlated with global cognitive performance (k = 53, r = −0.076 (95% CI: −0.126 to −0.027) *p* = 0.003), verbal memory (k = 36, r = −0.089 (95% CI: −0.148 to −0.029) *p* = 0.004), visual memory (k = 15, r = −0.036 (95% CI: −0.049 to −0.023) *p* = <0.001), working memory (k = 23, r = −0.065 (95% CI: −0.118 to −0.011) *p* = 0.018), and processing speed (k = 20, r = −0.082 (95% CI: −0.151 to −0.012) *p* = 0.022) but were not significantly associated with attention (k = 27, r = −0.027 (95% CI: −0.091 to 0.037) *p* = 0.412), reasoning (k = 37, r = −0.025 (95% CI: −0.096 to 0.045) *p* = 0.378), or language (k = 21, r = −0.052 (95% CI: −0.144 to 0.041) *p* = 0.271). Anti-inflammatory markers were not significantly associated with global cognitive performance (k = 5, r = 0.067 (95% CI: −0.069 to 0.201) *p* = 0.334), verbal memory (k = 3, r = −0.074 (95% CI: −0.240 to 0.097) *p* = 0.398), processing speed (k = 4, r = 0.125 (95% CI: −0.030 to 0.275) *p* = 0.113), or reasoning (k = 4, r = −0.002 (95% CI: −0.245 to 0.241) *p* = 0.985). In analyses restricted to patients with schizophrenia, proinflammatory markers were negatively correlated with visual memory (k = 9, r = −0.142 (95% CI: −0.241 to −0.040) *p* = 0.006) with no significant associations observed with global cognitive performance (k = 27, r = −0.036 (95% CI: −0.113 to 0.042) *p* = 0.370), verbal memory (k = 22, r = −0.072 (95% CI: −0.172 to 0.030) *p* = 0.168), working memory (k = 16, r = 0.002 (95% CI: −0.071 to 0.076) *p* = 0.947), attention (k = 16, r = −0.071 (95% CI: −0.164 to 0.022) *p* = 0.135) Processing speed (k = 12, r = −0.080 (95% CI: −0.171 to 0.013) *p* = 0.090), reasoning (k = 18, r = −0.026 (95% CI: −0.096 to 0.045) *p* = 0.476), or language (k = 16, r = −0.033 (95% CI: −0.137 to 0.072) *p* = 0.538).
Redddi Patlola *et al.* (2023)	Schizophrenia	Inflammatory biomarkers and cognitive dysfunction	IL-6 was significantly associated with attention and processing speed (k = 7, μ = -0.173, (95% CI: -0.302 to -0.045) *p* = 0.008), executive functioning (k = 8, μ = -0.181, (95% CI: -0.266 to -0.097) *p* < 0.001), verbal learning and memory (k = 7, μ = -0.169, (95% CI: -0.307 to -0.032) *p* = 0.016), and working memory (k = 6, μ = -0.136, (95% CI: -0.270 to -0.003) *p* = 0.046), but not with visual learning and memory (k = 4, μ = -0.08, (95% CI: -0.227 to 0.065) *p* = 0.279). TNF-α levels were significantly associated with attention and processing speed (k = 9, μ = -0.198, (95% CI: -0.341 to -0.055) *p* = 0.007), executive functioning (k = 9, μ = -0.188, (95% CI: -0.324 to -0.052) *p* = 0.007), and visual learning and memory (k = 4, μ = -0.38, (95% CI: -0.538 to -0.222) *p* < 0.001) but not with verbal learning and memory (k = 7, μ = -0.118, (95% CI: -0.324 to 0.089) *p* = 0.610) or working memory (k = 6, μ = -0.15, (95%CI:-0.367 to 0.067) *p* = 0.175). IL-1β levels significantly associated with attention and processing speed (k = 4, μ = -0.372, (95% CI: -0.619 to -0.125) *p* = 0.003), visual learning and memory (k = 3, μ = -0.454, (95% CI: -0.627 to -0.282) *p* < 0.001), and working memory (k = 3, μ = -0.476, (95% CI: -0.649 to -0.303) *p* < 0.001). CRP levels significantly associated with attention and processing speed (k = 9, μ = -0.299, (95% CI:-0.494 to -0.103) *p* = 0.003), verbal learning and memory (k = 7, μ = -0.278, (95% CI: -0.356 to -0.20) *p* < 0.001), visual learning and memory (k = 5, μ = -0.311, (95% CI: -0.538 to -0.084) *p* = 0.007), working memory (k = 6, μ = -0.168, (95% CI: -0.315 to -0.022) *p* = 0.024), but not executive functioning (k = 5, μ = -0.159, (95% CI: -0.502 to 0.184) *p* = 0.364).
Zheng *et al.* (2020)	Alzheimer’s disease and controls	Cortisol secretion and cognitive decline	Compared to cognitively-normal controls, Alzheimer’s disease patients showed significantly increased morning cortisol levels when measured in blood (k = 38, g = 0.422 (95% CI: CI: 0.289 to 0.556) *p* < 0.001), saliva (k = 5, g = 0.540, (95% CI: 0.276 to 0.804) *p* < 0.001) and CSF (k = 5, g = 0.565 (95% CI: 0.198 to 0.931) *p* = 0.003). In contrast, MCI patients showed no differences in cortisol levels when measured in blood (k = 5, g = 0.002 (95% CI: -0.162 to 0.167) *p* = 0.978) or saliva (k = 10, g = 0.106 (95% CI: -0.047 to 0.258) *p* = 0.174) when compared to cognitively-normal controls but showed elevations in CSF cortisol (k = 4, g = 0.309 (95% CI: 0.125 to 0.492) *p* = 0.001).

**Abbreviations:** k: number of effect sizes included in analysis; g: Hedges g effect size; SMD: standardised mean difference; CI: confidence interval; CRP: C-reactive protein; IL: interleukin; IFN: interferon; TNF: tumour necrosis factor; CSF: cerebrospinal fluid; MCI: mild cognitive impairment.

**Table 5:** Meta-analyses examining associations between childhood trauma and neuroimaging measures in clinical and non-clinical populations.

**Author**	**Population**	**Measure**	**Findings**
Nikolova *et al.* (2021)	Psychiatric disorders	Gut microbiota alterations across psychiatric disorders	Analysis of alpha diversity showed a significant decrease in richness (number of species) across all observed species compared to controls for pooled analysis of all psychiatric disorders (k = 20, SMD = −0.26 (95% CI: −0.47 to −0.06) *p* = 0.01) and for sub-analyses examining bipolar disorder (k = 3, SMD = –0.61 (95% CI: –1.19 to –0.03), but no significant differences in sub-analyses comparing patients with MDD (k = 6, SMD = –0.16 (95% CI: –0.58 to 0.27) or schizophrenia and psychosis (k = 4, SMD = –0.04 (95% CI: –0.31 to 0.24) with controls. Studies examining Chao1 showed a significant decrease relative to controls when data were pooled across all psychiatric disorders (k = 26, SMD = −0.5 (95% CI: −0.79 to −0.21) *p* = .001), and significant decreases for bipolar disorder (k = 4, SMD = –0.53 (95% CI: –1.01 to –0.05) and anorexia nervosa (k = 4, SMD = –0.86 (95% CI: –1.52 to –0.21). No significant differences were observed for sub-analyses comparing controls with MDD (k = 6, SMD = –0.34 (95% CI: –1.08 to 0.40) or schizophrenia and psychosis patients (k = 7, k = –0.58 (95% CI: –1.29 to 0.12), Compared to controls, no significant differences in the Shannon index were observed across all psychiatric disorders (k = 29, SMD = −0.12 (95% CI: −0.27 to 0.03) *p* = .11), MDD (k = 11, SMD = –0.28 (95% CI: –0.62 to 0.06), bipolar disorder (k = 4, SMD = 0.09 (95% CI: –0.43 to 0.62), or schizophrenia and psychosis (k = 8, SMD = –0.02 (95% CI: –0.20 to 0.17). No differences compared to controls in the Simpson index were observed across all psychiatric disorders (k = 11, SMD = 0.04 (95% CI, −0.13 to 0.21) *p* = .66) MDD (k = 5, SMD = 0.14 (95% CI: –0.14 to 0.43), or bipolar disorder (k = 3, SMD = –0.03 (95% CI: –0.34 to 0.28). Phylogenetic diversity was significantly reduced when data from all psychiatric patients were compared with controls (k = 10, SMD = −0.24 (95% CI, −0.47 to −0.0012) *p* = .049), but not in sub-analyses examining MDD (k = 4, SMD = –0.42 (95% CI: –0.96 to 0.13) or schizophrenia and psychosis (k = 3, SMD = –0.01 (95% CI: –0.22 to 0.20).
Safadi *et al.* (2022)	Severe mental illness	Biomarkers of gut dysbiosis differences in severe mental illness	A combined analysis of BPD and MDD samples showed significant increases in zonulin (k = 4, SMD = 0.97 (95%Cl: 0.10 to 1.85) *p* = 0.03) relative to controls. Antibodies to endotoxins were significantly elevated in BPD (k = 2, SMD = 0.72 (95% CI: 0.54 to 0.90), MDD (k = 2, SMD = 0.77 (95% CI: 0.43 to 1.12), but not schizophrenia (k = 3, SMD = 1.16 (95% CI: -0.48 to 2.81). Soluble CD14: significantly elevated relative to controls in BPD (k = 3, SMD = 0.61 (95% CI: 0.15 to 1.07) but not schizophrenia (k = 3, SMD = 0.29 (95% CI: -0.24 to 0.82). Alpha-1-antitrypsin was significantly elevated in MDD (k = 3, SMD = 0.94 (95% CI: 0.35 to 1.53) and schizophrenia (k = 3, SMD = 1.79 (95% CI: 1.16 to 2.42) relative to controls. Intestinal fatty-acid binding protein showed no significant differences in comparisons between MDD and controls (k = 3, SMD = 0.30 (95% CI: -0.21 to 0.82).

**Abbreviations:** k: number of effect sizes included in analysis; SMD: standardised mean difference; CI: confidence interval; *r:* correlation coefficient; MDD: major depressive disorder; BPD: bipolar disorder.

**Table 6:** Meta-analyses examining treatment effects.

**Author**	**Population**	**Measure**	**Findings**
Capuzzi *et al*. (2017)	Medication-naïve FEP	Cytokines levels following 4 weeks of antipsychotic treatment	Among medication-naïve FEP patients, levels of IL-2 (k = 2, SMD = -0.47 (95% CI: −0.87 to −0.07) *p* = 0.023) and IL-6 (k = 4, SMD = -0.51 (95% CI: −0.92 to −0.11) *p* = 0.012) were significantly reduced following 4 weeks of antipsychotic treatment. No differences observed in levels of IL-1β (k = 4, SMD = -0.19 (95% CI: -0.66 to 0.28) *p* = 0.437), IL-I7 (k = 2, SMD = -0.57 (95% CI: -1.22 to 0.09) *p* = 0.088), IFN-γ (k = 2, SMD = -0.42 (95% CI: -0.87 to 0.03) *p* = 0.068) or TNF-α (k = 4, SMD = 0.03 (95% CI: -0.16 to 0.22) *p* = 0.745) were observed.
Jeppesen *et al*. (2020)	Schizophrenia and other psychotic disorders	Response to immuno-modulating medications	Treatment with antipsychotics and anti-inflammatory treatment was associated with a significant improvement in total psychopathology (k = 55, SMD = -0.29 (95% CI: -0.40 to -0.19) *p* < 0.001), positive psychotic symptoms (k = 56, SMD = -0.13 (95% CI: -0.21 to -0.05) *p* = 0.002), negative symptoms (k = 55, SMD = -0.23 (95% CI: -0.35 to -0.12) *p* < 0.001), general psychopathology (k = 44, SMD =-0.16 (95% CI: -0.26 to -0.06) *p* = 0.002), and symptom severity (k = 13, SMD =-0.22 (95% CI: -0.40 to -0.03) *p* = 0.024) compared to antipsychotic medication and placebo, but was not associated with a significant improvement in functioning (k = 7, SMD =0.14 (95% CI: -0.13 to 0.42) *p* = 0.312).
Lombardo *et al*. (2019)	Mood disorders (major depression with and without psychotic features and bipolar)	Baseline cortisol levels and response to anti-glucocorticoid treatment	In combined analysis of antiglucocorticoid treatments combined, treatment responders showed no differences in baseline cortisol levels compared to non-responders (k = 16, SMD=−0.025 (95% CI: −0.173 to 0.124), *p* = 0.747). In sub-analyses examining cortisol synthesis inhibitors, responders showed significantly higher peripheral baseline cortisol levels compared with non-responders (k = 4, SMD=0.421 (95% CI: 0.010, 0.833), *p =* 0.045). In sub-analyses examining GR antagonists, no differences in cortisol levels were observed in comparisons between responders and non-responders (k = 12, SMD= −0.092 (95% CI: −0.251, 0.068) *p =* 0.261).
Minichino *et al*. (2021)	Schizophrenia and other psychotic disorders	Response to gut-microbiome targeted interventions	Compared to placebo, adjunctive treatment with sodium benzoate was associated with significant improvement in negative symptoms (k = 2, SMD = −0.63 (95% CI: −1.03 to −0.23) *p* < 0.001), positive symptoms (k = 2, SMD = −0.94 (95%CI −1.35 to −0.53) *p* < 0.001)), and total symptoms (k = 2, SMD = −0.68 (95% CI: −1.08 to −0.28) *p* = 0.001). Analyses for negative symptoms showed that neither D-cycloserine (k = 10, SMD = −0.16 (95% CI: −0.40 to 0.08) *p* = 0 .20) nor minocycline (k = 7, SMD = −0.35 (95% CI −0.70 to 0.00) *p* = 0.05) were not significantly superior to placebo.

**Abbreviations:** FEP: first-episode psychosis; k: number of effect sizes included in analysis; SMD: standardised mean difference; CI: confidence interval; CRP: C-reactive protein; IL: interleukin; IFN: interferon; TNF: tumour necrosis factor; GR: glucocorticoid receptor.

## LIST OF ABBREVIATIONS

ACTH: Adrenocorticotropic Hormone
AUC: Area Under the Curve
BDNF: Brain-Derived Neurotrophic Factor
CAR: Cortisol Awakening Response
CHR-P: Clinical High-Risk for Psychosis
CI: Confidence Interval
CRH: Corticotrophin-Releasing Hormone
CRP: C-Reactive Protein
DHEAs: Sulphate Dehydroepiandrosterone
EMA: Ecological Momentary Assessment
ESM: Experience Sampling Methods
FEP: First-Episode Psychosis
FHR: Familial High-Risk
GFM: Germ-Free Mice
GRs: Glucocorticoid Receptors
HPA: Hypothalamic-Pituitary-Adrenal Axis
IFN: Interferon
IL: Interleukin
MIA: Maternal Immune Activation
mRNA: Messenger Ribonucleic Acid
MRs: Mineralocorticoid Receptors
NICE: National Institute for Health and Care Excellence
NMDA: N-methyl-D-aspartate
OR: Odds Ratio
PLEs: Psychotic-Like Experiences
PTSD: Post-Traumatic Stress Disorder
*r*: Pearson's Correlation Coefficient
rCBF: Resting Cerebral Blood Flow
RCT: Randomised Controlled Trial
rs-FC: Resting-State Functional Brain Connectivity
SPD: Schizotypal Personality Disorder
TGF: Transforming Growth Factor
TNF: Tumor Necrosis Factor
